# Investigating microbial and environmental drivers of nitrification in alkaline forest soil

**DOI:** 10.1093/ismeco/ycae093

**Published:** 2024-07-11

**Authors:** Lianna Poghosyan, Laura E Lehtovirta-Morley

**Affiliations:** School of Biological Sciences, University of East Anglia, Norwich NR4 7TJ, United Kingdom; School of Biological Sciences, University of East Anglia, Norwich NR4 7TJ, United Kingdom

**Keywords:** ammonia-oxidizing microorganisms, nitrification, soil pH, unfertilized soil, microbial abundance

## Abstract

Ammonia oxidation is a key step in the biogeochemical cycling of nitrogen, and soils are important ecosystems for nitrogen flux globally. Approximately 25% of the world’s soils are alkaline. While nitrification has been studied more extensively in agricultural alkaline soils, less is known about natural, unfertilized alkaline soils. In this study, microorganisms responsible for ammonia oxidation and several environmental factors (season, temperature, ammonia concentration, and moisture content) known to affect nitrification were studied in an alkaline forest soil with a pH ranging from 8.36 to 8.77. Ammonia-oxidizing bacteria (AOB), ammonia-oxidizing archaea, and comammox were present, and AOB belonging to genera *Nitrosospira* and *Nitrosomonas*, originally comprising <0.01% of the total bacterial community, responded rapidly to ammonia addition to the soil. No significant difference was observed in nitrification rates between seasons, but there was a significant difference between *in situ* field nitrification rates and rates in laboratory microcosms. Surprisingly, nitrification took place under many of the tested conditions, but there was no detectable increase in the abundance of any recognizable group of ammonia oxidizers. This study raises questions about the role of low-abundance microorganisms in microbial processes and of situations where zero or very low microbial growth coincides with metabolic activity. In addition, this study provides insights into nitrification in unfertilized alkaline soil and supports previous studies, which found that AOB play an important role in alkaline soils supplemented with ammonia, including agricultural ecosystems.

## Introduction

Nitrification is the sequential oxidation of ammonia to nitrate via nitrite and is an important part of the global biogeochemical nitrogen cycle in many natural and engineered environments. This environmentally widespread process is carried out exclusively by three functionally similar, yet evolutionarily distinct groups of microorganisms: canonical ammonia-oxidizing bacteria (AOB) (noncomammox microorganisms), ammonia-oxidizing archaea (AOA), and comammox *Nitrospira*. Nitrogen availability is one of the main limiting factors for the primary productivity of terrestrial plants [1, 2]. The amount of reactive, bioavailable nitrogen in the environment continues to increase because of anthropogenic activities [3–5]. The use of organic and inorganic nitrogen fertilizers in agriculture leads to emissions of the global-warming and ozone-depleting gas, nitrous oxide (N_2_O) to the atmosphere [6–9]. Another consequence is that much of the excess nitrate enters aquatic environments, where it causes eutrophication and consequent perturbations to the ecosystem such as algal blooms and hypoxia [10–12].

For over a century, AOB were assumed to be the only chemolithoautotrophic microorganisms, which can generate energy from ammonia oxidation to nitrite. However, the advancement of high-throughput sequencing and novel isolation techniques led to the identification of AOA [13, 14] and two clades (A and B) of complete ammonia oxidizing *Nitrospira* (comammox) [15, 16]. The known nitrifiers are ubiquitous in nature where they coexist. However, the abundance and diversity of nitrifiers, as well as environmental nitrification rates, vary widely across different ecosystems [17–20].

Alkaline soils cover approximately 25% of Earth’s surface and are common in semiarid and arid climates [21]. Nitrification in alkaline conditions is typically studied in agricultural soils, rather than in natural, unmanaged soils. Alkaline soils often contain calcium carbonate, which is also used in agricultural practice (liming) to increase the soil pH and fertility. Volatilization of ammonia at high pH and the related nitrogen loss present an additional challenge to agriculture in alkaline soils [22]. Both AOA and AOB are found in alkaline agricultural soils, including purple soil, silt loam, and clay loam soils, with some indications that AOB contribute more than AOA to nitrification and N_2_O emissions in these ecosystems [7, 23–26]. The role of comammox *Nitrospira* in alkaline conditions has not been explored to the same extent as the roles of AOA and AOB. Comammox *Nitrospira* are present and active in alkaline soils [27], and recently nitrification by comammox *Nitrospira* in saline–alkaline lakes with pH of 11 was reported [28], suggesting that comammox bacteria could be important microbial players in habitats with high pH.

Ammonia oxidation kinetics and substrate affinities are known as key factors determining the habitat preferences of different nitrifying groups of microorganisms [29–31]. Recent studies suggest that there is a level of overlap in the affinities of terrestrial AOB and AOA to ammonia [32]. Several studies indicate that in soil, AOA and comammox thrive under conditions where ammonia is supplied through mineralization, while AOB dominate when ammonia concentrations are high, typically in N-amended soils [33–39]. However, multiple environmental factors (land-use, soil type, pH, ammonia availability, temperature) can together play an important role in niche separation of nitrifying microorganisms [40, 41]. Since ammonia is a weak base that exists in both protonated (NH_4_^+^) and unprotonated (NH_3_) forms (pKa 9.27) and since the substrate for ammonia monooxygenase is NH_3_ [32, 42], environmental pH can influence the distribution and activity of ammonia oxidizers in soil environments by decreasing bioavailable ammonia concentrations as pH decreases [20, 41]. Although many groups of aerobic ammonia oxidizers are ubiquitous [43–47], the literature suggests that AOA and clade B comammox *Nitrospira* might be better adapted to acidic conditions than clade A comammox and AOB [33, 37, 48–51]. While nitrification in acidic soils has been studied extensively over the last few decades, nitrification in alkaline conditions, particularly in natural habitats, has been investigated to a lesser extent. The aim of this study was to determine the abundance, diversity, and activity of ammonia oxidizers in alkaline forest soil. Furthermore, the aim was to investigate the influence of environmental factors, including temperature, moisture content and ammonia availability, on the nitrifying microbial community and net nitrification rates in the field and using laboratory microcosms.

## Material and methods

### Sampling site and soil properties

The study site was a broadleaf woodland in calcareous soils near Thetford, UK (52°24′50.0″N, 0°51′45.0″E). According to the UK Soil Observatory (https://www.ukso.org/), the chalky soil texture in Thetford Forest soil is light to medium sandy with a pH range of 4.4–8.2. The experiments were established in January 2021 and April 2021. The pH at the alkaline site was 8.36 in winter and 8.7 in the spring. Three 200–400 g topsoil samples were collected from 0–10 cm depth, sieved through a 2-mm mesh to remove stones and plant roots and mixed. The fresh soil samples were subsampled for pH, inorganic N, and soil moisture measurements. To measure gravimetric soil moisture content, 10 g of soil was weighed, oven dried at 105°C for 24 h until there was no further mass loss, and reweighed. The moisture content is expressed as % of water (by weight) per dry soil weight (d.s.w.). Soil pH was measured in triplicate at a water to soil ratio of 2:1 (w/v) with a glass electrode pH meter (Benchtop 3505 pH meter, Jenway). To measure the inorganic N levels in soils, a subsample of 1 g of soil was extracted with 10 ml of 2 M KCl. Soil NH_4_^+^, NO_2_^−^, and NO_3_^−^ were determined colorimetrically by modified indophenol blue method [52] and VCl_3_/Griess reaction [53, 54], respectively.

### Field nitrification rates—*in situ* buried bags

In this study seasonal net nitrification rates, as defined by accumulation of total NO_3_^−^ over time, at the field site were determined by field incubations using a short-term *in situ* buried bag approach [55]. Triplicate subsamples (~30 g) from the fresh, sieved composite soil sample were placed in separate grip seal polythene bags. Polyethylene is permeable to oxygen and carbon dioxide [55]. Sample bags were sealed during incubation to prevent the exchange of dissolved nitrogen in the water while allowing exchange of gases. Sample bags were buried in the field at a depth of approximately 10–15 cm and incubated *in situ*. Sample bags were removed after 0, 10, 20, and 30 days of incubations, and stored at −20°C for subsequent analyses. Temperature was inferred from UK Met Office data.

### Laboratory soil microcosms

The influence of temperature, moisture content, and ammonia availability on the net nitrification rates were determined by aerobic incubations under controlled laboratory conditions. Soil microcosms were established in 120-ml serum bottles containing 10 g of soil and incubated in the dark. For each treatment, microcosms were destructively sampled in triplicate at 0, 10, 20, or 30 days. Laboratory microcosms testing the effect of moisture and ammonia concentration were performed using soil sampled in spring. For temperature perturbation experiments, the native soil sampled both in spring and in winter was incubated at 30°C, and net nitrification rates were compared to field incubations. To determine the influence of moisture content on the net nitrification rates, the moisture content was adjusted to 16% (w/w) gravimetric water content with Milli-Q water. Soils were not dried at any stage because drying may impact microbial communities. Therefore, moisture adjustment was performed by adding water into the native soil as it was at the time of sampling. Native (12% w/w) and moisture-adjusted soil was then used for constructing microcosms and incubated at room temperature (21°C). To assess the influence of ammonia availability on the net nitrification rates, soil samples were amended with 20 or 200 mg (final concentration) NH_4_^+^-N kg^−1^ d.s.w., supplied as NH_4_Cl, and the final moisture content was adjusted to 16% (w/w). Following preincubation at 4°C for 24 h, microcosms were constructed and incubated at room temperature (21°C). At intervals of 4–5 days microcosms amended with 20-mg NH_4_^+^-N kg^−1^ d.s.w. were supplemented with ammonium chloride solution to restore the starting ammonium concentrations. The 200-mg NH_4_^+^-N kg^−1^ d.s.w. microcosms were only supplemented on day 17. The volume of ammonium solution added to the lower concentration ammonia-amended microcosms gradually increased the final moisture content to 18%. To prevent water evaporation, bottles were sealed with 20-mm moulded butyl septa (Thermo Fisher Scientific B.V.). Aerobic conditions were maintained by opening and resealing the bottles at intervals of 4–5 days. Following destructive sampling at each timepoint soils were stored at −20°C for subsequent analyses. As the nitrate accumulation remained approximately the same throughout the incubations, the net nitrification rates for both *in situ* incubations and laboratory microcosms were calculated at the end of the experiment by subtracting T = 0 measurement from the final nitrate concentration and dividing by the number of days.

### DNA extraction and quantitative PCR analyses

At each timepoint, DNA was extracted from 0.25 g wet weight soil using the DNeasy® PowerSoil® Pro Kit (Qiagen, Benelux BV) according to the manufacturer’s instructions. Gene abundances were quantified by quantitative PCR (qPCR) with a SensiFAST SYBR No-ROX Kit (Bioline, London, UK) on a StepOnePlus instrument (Applied Biosystems, Waltham, MA, USA). Reaction mixtures of 15 μl contained 7.5 μl SensiFAST Master Mix, 1 μl of each primer (~660 nM final concentration), and 5 μl of genomic DNA (2 ng/μl). qPCR standards of AOA and AOB were generated by PCR using “*Ca. Nitrosocosmicus franklandus* C13” and *Nitrosomonas europaea* ATCC 19718 genomic DNA as templates (Table S1). qPCR standards for comammox were generated by PCR using “*Ca. Nitrospira kreftii*” (enrichment culture) [56] genomic DNA as template and cloning the amplicon into pGEM-T Easy Vector System (Promega, Madison, WI, USA) (Table S1). The cloned plasmids containing the “*Ca. N. kreftii*” *amoA* sequence were amplified with M13 primers, and PCR products were purified with ExoSAP-IT kit (Applied Biosystems, Foster City, CA, USA). All the standards were generated in triplicates by 10-fold serial dilutions (10^1^–10^7^ gene copies/reaction). Gene abundances were quantified with absolute quantification method against an internal standard calibration curve of each target gene. The genomes of almost all AOA contain a single copy of *amoA*, but AOB genomes may contain up to three copies of *amoA*. The AOB qPCR values were therefore normalized by dividing by 2.5, which is the average number of *amoA* copies in known AOB genomes [57]. Most AOA also contain a single 16S rRNA gene, and comammox contain a single *amoA* gene, and thus no normalization was done for these qPCR assays. When interpreting the absolutely abundances, it is important to note that the cell size, protein content, and kinetic properties vary between different ammonia oxidizers [32]. The *Thaumarchaeota* 16S rRNA and AOB *amoA* genes were amplified under the same conditions using primers described in Table S1: initial denaturation at 95°C for 15 min followed by 40 cycles of denaturation at 94°C for 10 s, combined annealing and extension at 62°C for 30 s, and fluorescence reading at 72°C for 10 s. For comammox *amoA* the same conditions were used, except for annealing and fluorescence reading at 64°C for 30 s. Amplification specificity was confirmed by melt curve analysis and by running the qPCR products on agarose gels. The correlation coefficient (*r^2^*) for each standard curve was >0.99. Amplification efficiencies for archaeal 16S rRNA, AOB, and comammox *amoA* assays were 96.6%, 87.2%, and 92.7%, respectively.

### 16S rRNA gene amplicon sequencing and microbial profiling

Small subunit rRNA gene amplicons were sequenced by Illumina MiSeq Next Generation Sequencing (Macrogen, Korea). For amplicon sequencing the V4 hypervariable region was PCR amplified using bacterial Bac341F [58] and Bac806R [59] and archaeal Arch349F and Arch806R [60]. These primers have been used extensively for previous studies of bacteria and archaea and have been validated to be specific. Amplicon sequence variants (ASVs) were assigned using the DADA2 package v1.24.0 [61] in R v4.2.1 [62]. Briefly, the demultiplexed 16S rRNA gene sequences were primer-removed and processed by trimming to 280 and 250 base pairs for the forward and reverse reads, respectively, using the filterAndTrim command with standard parameters for quality trimming, estimated error, and ambiguous nucleotides (maxN = 0, maxEE = c(2,2), truncQ = 2, rm.phix = TRUE). Processed reads were error-corrected, dereplicated, and merged, and ASV taxonomy was assigned using the SILVA v138 database [63]. The bacterial dataset was rarefied to 28 000 reads and the archaeal dataset to 50 000, followed by diversity analysis using the phyloseq v1.40.0 package in R [64]. Sequences have been deposited in NCBI under Bioproject accession number PRJNA1063581.

### Phylogenetic trees

Reference sequences were obtained from NCBI and aligned using BioEdit v7.2.5 [65]. Positions containing gaps were removed and phylogenetic analyses performed in MEGA v7.0.14 [66]. Phylogenetic trees were constructed using the maximum likelihood method based on the Tamura-Nei model with 100 bootstrap replicates.

### Statistical analysis

Homogeneity of variance for each treatment was tested by using the Levene test and normality by the Shapiro–Wilk test. Significant changes in nitrification rates for different treatments was independently assessed by one-way analysis of variance followed by Tukey Honest Significant Differences (HSD) post hoc test for multiple pairwise comparison between the means of groups. The changes in gene abundance during the incubation period were assessed by paired samples *t*-tests. Unpaired two sample *t*-tests were used to compare the means of two independent groups. All statistical analyses were conducted using R statistical language version 4.2.1 and the R standard libraries [62].

## Results

### Soil physicochemical properties

The pH of the soil collected from Thetford Forest remained relatively consistent in both seasons (winter and spring) in this study, with a narrow range of 8.36–8.77. The gravimetric soil moisture content was 9.0% and 12.5% (g g^−1^ of dry soil) in January and April, respectively. The inorganic nitrogen was dominated by NO_2_^−^ + NO_3_^−^−N (NO_x_^−^-N) with levels ranging from 2.1 to 2.7 mg kg dry soil^−1^. The NH_4_^+^-N was below the detection limit (0.6 mg kg dry soil^−1^) in January but in April was found to be 1.4 mg kg dry soil^−1^.

### Nitrification rates

**Figure 1:**
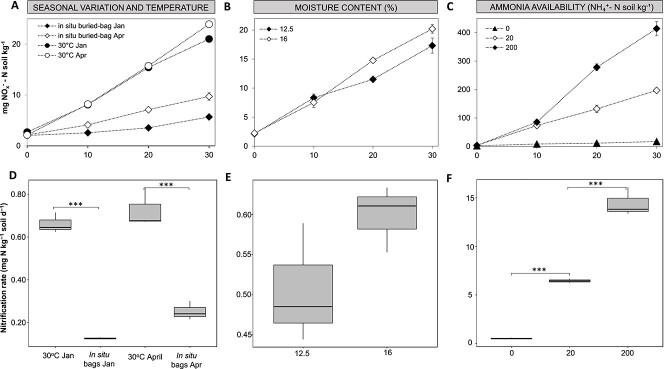
The NO_x_^−^ production (A–C) and nitrification rates (D–F) in soil microcosms following application of different treatments (temperature (A, D), moisture (B, E), ammonia concentration (C, F)), and comparing nitrification rates between seasons. Laboratory microcosms testing the effect of moisture and ammonia concentration (panels B, C, E, and F) were carried out using soil sampled in spring. The gravimetric water content in the ammonia-amended microcosms (C, F) was 16%. Additional data indicating the times at which ammonium was added to the microcosms are shown in the Supplementary Material. Data were presented as mean values with standard errors of the mean (*n* = 3). Nitrification *in situ* and in unamended microcosms depends on the production of ammonium from mineralization. As the soil is not water logged, and presumed to be aerobic, it is expected that little or no denitrification takes place under the tested conditions. Nitrification occurred throughout the incubations at approximately the same rate.

Microcosms were set up to compare seasonal effects and the differences between net nitrification *in situ* at ambient temperature and laboratory microcosms incubated at 30°C. *In situ* nitrification rates were assessed by the buried bag approach, which has been previously used for measuring nitrogen mineralization and nitrification in soils [67, 68]. In this approach, polyethylene bags containing soil are sealed and placed underground, and nitrate accumulates as a result of mineralization and nitrification because nitrate is not available for plant uptake or leaching. We hypothesized that nitrification rates and microorganisms responsible would differ between *in situ* and laboratory incubations. Soil net nitrification rates in all the microcosms were determined by measuring net accumulation of NO_x_-N over a period of 30 days. Field net nitrification measured *in situ* in buried bags occurred at a rate of 0.12 and 0.29 mg N kg^−1^ soil day^−1^ during the winter and spring seasons, respectively (Fig. 1A). While the total net production of NO_x_-N over the time period of the experiment was higher in spring (9.7 ± 0.7 mg N kg^−1^ soil) than in winter (5.7 ± 0.08 mg N kg^−1^ soil), the difference in the *in situ* nitrification rates was no statistically significant (*P* = .09, Tukey HSD test). The nitrification rates (0.66–0.72 mg N kg^−1^ soil day^−1^) in the 30°C laboratory microcosms were significantly higher (*P* < .01*,* Tukey HSD test) than in the field microcosms, which experienced temperatures ranging 5–13°C in winter and 9–18°C in spring (Fig. 1).

To investigate the effects of moisture and ammonia concentration on nitrification rates, laboratory microcosms were amended with either water or ammonia. The soil moisture content did not have a significant influence (*P* = .132, two-sample *t*-test) on the nitrification rates. The nitrification rates were 0.50 and 0.59 mg N kg^−1^ soil day^−1^ in the microcosms incubated with 12.5% (w/w) and 16% (w/w) gravimetric water content, respectively. Preliminary experiments were conducted to determine the optimal frequency of ammonium addition to the soils amended with 20 and 200 mg NH_4_^+^-N kg^−1^ soil (Supplementary Fig. S1). In the microcosms amended with 20 mg NH_4_^+^-N kg^−1^ soil, ammonium was fully oxidized on day 4, whereas in amendments with 200 mg NH_4_^+^-N kg^−1^, ammonium was fully oxidized only after 17 days (Supplementary Fig. S1). Therefore, microcosms were supplemented at intervals of either 4 or 17 days. Nitrification rates in the microcosms amended with 20 and 200 mg NH_4_^+^-N kg^−1^ occurred at significantly higher rates (6.4 and 14.4 mg N kg^−1^ soil day^−1^, respectively) (*P* < 0.01, Tukey HSD test).

### Abundance of AOA, AOB, and comammox genes

**Figure 2:**
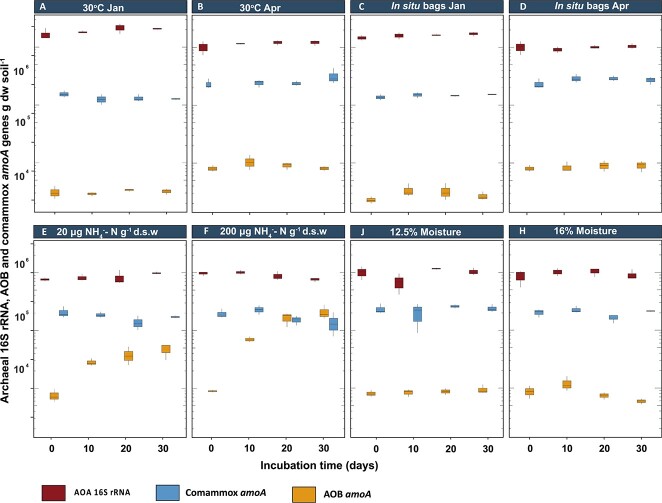
Abundance of AOA 16S rRNA, AOB, and comammox *amoA* genes in the alkaline forest soil microcosms in winter (January (Jan)) and spring (April (Apr)). Timepoints for the sampling are 0, 10, 20, and 30 days for all groups of nitrifiers. The error bars represent the standard error of the mean (*n* = 3).

To determine absolute abundances of different groups of nitrifiers, the gene abundances of AOA, AOB, and comammox *Nitrospira* genes were determined by quantitative PCR. In the freshly sampled forest soils, the number of AOA 16S rRNA genes decreased from 2.1 × 10^6^ ± 1.4 × 10^4^ copies g^−1^ dry soil to 1.01 × 10^6^ ± 8.5 × 10^4^ copies g^−1^ dry soil between winter and spring, respectively. The overall abundance of AOA 16S rRNA genes was significantly higher (*P* < .05) than that of AOB *amoA* and comammox *amoA* genes during both winter and spring (by approximately 2 and 1 orders of magnitude, respectively) (Fig. 2). Surprisingly, although nitrification occurred in all microcosms (Fig. 1), no increase in the abundance of nitrifiers was observed during the course of the incubation, except for the treatments with added ammonium (Fig. 2). In the ammonia-amended microcosms, the abundance of AOB *amoA* genes increased significantly (*P* < .05; paired samples *t*-test) during 30-day incubation period (Fig. 2E and F). The AOB *amoA* gene copy numbers between 0 and 30 days increased from 8 × 10^3^ ± 6.7 × 10^2^ to 4.7 × 10^4^ ± 4.6 × 10^3^ (*P* = .03) and 1.6 × 10^5^ ± 4.6 × 10^4^ (*P* = .04) per gram of dry soil for the 20 and 200 mg NH_4_^+^-N kg−^1^ soil, respectively. The addition of ammonia did not have any significant impact on the AOA 16S rRNA and comammox *amoA* gene abundances. No significant (*P* = .09) changes in AOB *amoA* gene abundance was observed in the microcosms with no added ammonium (Fig. 2J). The gene abundance of AOA 16S rRNA, and AOB and comammox *amoA* remained relatively stable over time in the *in situ* buried bags, and no significant changes were detected (*P >* .05) (Fig. 2). Similarly, in the laboratory incubations, the average copy numbers of the 16S rRNA and *amoA* genes did not increase in incubations at elevated temperature or moisture content over the course of the 30-day incubation period.

### Composition of the nitrifying community

**Figure 3:**
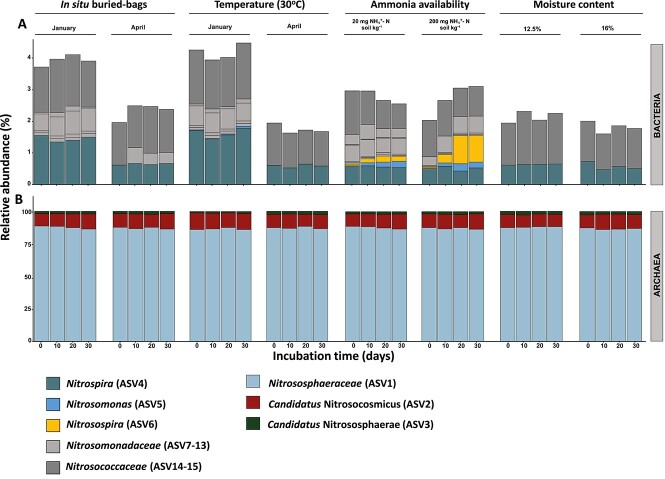
Average relative abundance of nitrifying communities of bacteria (A) and archaea (B) in the soil microcosms incubated under different *in situ* and laboratory conditions including temperature, ammonia concentration, and water content. ASVs with the relative abundance of <0.01% of the total reads are not included in the analysis. ASV numbers correspond to the phylogenies shown in Figs S2–S5.

The relative abundance and diversity of different nitrifying microorganisms were assessed by 16S rRNA gene amplicon analysis using separate primer sets for Archaea and Bacteria. 16S rRNA gene analysis showed that the archaeal community comprised AOA classified as *Nitrososphaerota* (Fig. 3). This is unsurprising as soil archaeal communities are often dominated by AOA. Analysis at the genus level allowed taxonomic identification of three groups of AOA, of which unclassified and uncultured *Nitrososphaeraceae* species (ASV1) represented ~84%–88% of the total archaeal community in all the soil samples (Figs 3 and S2). The representatives from genera *Candidatus* Nitrosocosmicus (ASV2) and *Nitrososphaera* (ASV3) comprised an average 12% and 2% of total archaeal sequences, respectively. However, the 16S rRNA gene amplicon sequencing analysis of soil microcosms incubated for 30 days revealed no significant changes in the overall archaeal community composition (Fig. 3).

Bacterial 16S rRNA gene analysis of the soil samples revealed the presence of bacteria belonging to sublineage II of genus *Nitrospira*, which contains nitrite oxidizers and comammox bacteria. The 0.01% was used as a cut-off to identify any nitrifying bacteria in the soil samples, and canonical AOB were only found in the ammonia-amended microcosm. In addition, several ASVs assigned to unclassified *Nitrosomonadaceae* (ASV7–ASV13) (Fig. S4) or unclassified *Nitrosococcaceae* (ASV14, ASV15) (Fig. S5) were detected. The phylogenetic placement of the unclassified ASVs suggests that they are affiliated with microorganisms not related to known AOB, i.e. heterotrophic bacteria such as *Chitinimonas* and *Limnobacter*, as well as sulphur-oxidizing bacteria belonging to γ-proteobacteria. The data therefore suggest that these sequences do not belong to ammonia oxidizers but are more likely to perform other functions (Figs S4 and S5). The absolute abundance of AOB was measured over time by qPCR in ammonia-amended microcosms (Fig. 2E and F), and sequences affiliated with representatives of the genera *Nitrosomonas* (ASV5) and *Nitrosospira* (ASV6) increased in abundance. In microcosms amended with 20 mg NH_4_^+^-N kg^−1^ soil, the abundances increased on average from <0.01% to 0.2% for each of the groups. Similarly, in microcosms amended with 200 mg NH_4_^+^-N kg-^1^ soil the abundance of ASV5 (*Nitrosomonas*) increased from <0.01% to 0.2%, while the relative abundance of the ASV6 (*Nitrosospira*) increased from 0.03% to 1% (Fig. 3). ASV5 (*Nitrosomonas*) was most closely related to *Nitrosomonas oligotropha* (Fig. S4), which among other AOB from Cluster 6 are found in a range of habitats including soils. ASV5 (*Nitrosomonas*) was also comparatively abundant at day 30 in the winter microcosms incubated at 30°C (0.07%) (Figs 3 and S4). The abundance of other unclassified *Nitrosomonadaceae* and *Nitrosococcaceae* did not change over the 30-day incubation period (Fig. 3). An unclassified *Nitrospira* (ASV4) was detected in all samples, and its abundance was higher in winter microcosms (~1.5%) than in spring microcosms (~0.6%) (Fig. 3). The abundance of ASV4 remained stable throughout the incubations (Figs 3 and S3). This seems to be consistent with qPCR data for comammox *Nitrospira*, although identification of comammox *Nitrospira* based on 16S rRNA analysis is not reliable and it was not possible to confirm whether ASV4 belongs to a comammox bacterium or a nitrite oxidizer [69].

## Discussion

This study set out to investigate the nitrifying microbial community and environmental factors affecting nitrification in an unamended alkaline forest soil. Although AOB, AOA, and comammox were present and nitrification, albeit at relatively low rates, occurred in all the incubations, surprisingly there was no observable increase in the abundance of ammonia oxidizers in most of the treatments in this study. It has been previously reported that microbes at very low relative abundance can perform important ecological functions [70–72] and microbes in the environment sometimes exhibit low or near-zero growth while maintaining activity [73, 74]. The use of RNA-based techniques in future studies may provide insights into microorganisms that are active, but not growing. In addition, studies rarely measure death rate or turnover of the microbial communities, although the mortality rates in soil microbial communities can be significant depending on the conditions [75]. Little is known about turnover of nitrifiers in soils, but in acidic nitrifying soil microcosms, treatment with nitrification inhibitors not only inhibited nitrification but caused a decline in the abundance of AOA [70]. Previous studies with nitrifying soil microcosms have found that growth of ammonia oxidizers was associated with nitrification driven by mineralization [71], but it is possible that this soil is in a steady state where the amount of ammonia produced from mineralization is sufficient to sustain the active nitrifying population, but not growth. Changing parameters such as temperature or moisture can affect nitrification rates either by directly or by influencing mineralization rates, and thus the substrate availability, in treatments without ammonia amendments [72]. Consistent with the importance of mineralization for nitrification, several previous soil microcosm studies have reported growth of ammonia oxidizers when no additional ammonia was applied to the microcosms, which is in contrast to our study [76].

The exception to the lack of observable increase in abundance was in the treatments amended with additional ammonia, where AOB affiliated to genera *Nitrosospira* and *Nitrosomonas*, originally comprising <0.01% of the total bacterial community, increased in abundance. The ability of AOB to rapidly respond to elevated ammonia concentrations is extensively reported in literature [26, 36, 77]. Under laboratory conditions, the growth rates of AOB typically exceed those of AOA [30]. This may play a role in the competition between AOA and AOB in soil and explain the observed rapid response of AOB after the ammonia addition. However, more surprisingly the abundance of AOB, but not of AOA, also increased in the presence of 20 ppm ammonia in this study. This is in contrast to a previous study on differential ammonia concentrations in neutral pH agricultural soil, which found that AOA responded more strongly to 20 ppm and AOB to 200 ppm ammonia [36]. While the difference between the studies cannot be attributed to a single factor, it is interesting to note that ammonia, rather than ammonium, is the preferred substrate for ammonia oxidizers. As the availability of ammonia is dictated by pH (with pK_a_ = 9.27 for ammonia), the amount of bioavailable ammonia increases with increasing pH. The increase in ammonia availability may be favourable for AOB over AOA, as previous studies have found that AOB thrive in conditions with high ammonia concentrations [30, 36].

One of the challenges of laboratory-based experiments is relating the information back to the environment. These challenges include spatiotemporal dynamics in soils, plant–soil–microbial feedbacks, and sampling disturbances [78] (and references therein). For example, potential nitrification rate assays can be challenging to interpret due to selection of specific members of the microbial community by the experimental conditions [79]. Additional considerations for linking *in situ* and laboratory studies include comparisons of processes across scales, coupling of processes and multiple factors influencing the microbiome [78]. To explore the differences between the soil environment and the laboratory experiments, this study compared nitrification rates using *in situ* incubations and laboratory microcosms. The nitrification rates were significantly higher in the laboratory microcosms. As the field incubations took place under ambient temperatures and the laboratory microcosms at 30°C, temperature may contribute to this difference as has been previously reported for nitrification rates in soil [80, 81]. In this study, net nitrification as opposed to gross nitrification rates were measured, meaning that other nitrogen cycling processes such as assimilation and denitrification may have also occurred. The gross nitrification rates can be determined using ^15^N tracers; however, the ^15^N pool dilution method also has caveats including potential for cross-feeding during long incubations and the necessity to introduce additional nitrogen in the studied ecosystem, which particularly in the case on low nitrogen non-agricultural soils, may impact the microbial community and processes. ^15^N tracer experiments are often carried out over hours or days, whereas the microcosm incubation in this study lasted 30 days, which would make cross-feeding of ^15^N more likely if ^15^N tracers had been added. Nevertheless, ^15^N tracer experiments would be valuable for future studies to enable comparisons between net and gross nitrification rates and to evaluate the role of other microbial processes in the nitrogen flux.

AOA and comammox were present in the alkaline forest soil in this study, but the temperature, water content, or ammonia addition had no observable effects on the relative or absolute abundance of these microorganisms. AOA were affiliated to *Nitrososphaeraceae* and specifically genera *Candidatus* Nitrosocosmicus and *Nitrososphaera*, which have been previously reported in soils over a wide range of pH, including alkaline soils [82]. The abundance of AOA was greater in winter than in summer, which implies a potential seasonal effect. Climate, and especially the aridity index, has been identified as an important factor influencing ammonia oxidizing microbial communities and nitrification rates in soils [83]. As the moisture level changed only slightly with seasons at our study site, it is possible that the seasonal changes in this study were not sufficiently strong to greatly impact nitrifying communities or process rates. In this study, the increase in temperature resulted in an increase in nitrification rate. Previous studies have suggested temperature and moisture to be important factors for the activity of AOA and comammox [25, 80]. Generally, AOA tend to favour higher temperatures over AOB [25]. In the alkaline forest soil, AOA outnumbered AOB by two orders of magnitude. Some previous studies on agricultural alkaline soils have found that the AOA abundance was greater than that of AOB [7, 23], while other studies found the opposite [24]. Nevertheless, this comparison is complicated by the fact that the abundance of AOB would typically be higher in fertilized soil as opposed to unfertilized soil such as the one in this study. Soil pH is also known to be an important factor selecting for specific species of nitrifiers, and the AOA and AOB detected in this study were related to species previously detected in soil environments, including alkaline soils. Activity of comammox has been previously reported to be affected by moisture content, and consequently availability of oxygen, in the soil [84]. The alkaline forest soil in this study contained naturally low ammonia concentrations, and these conditions are often favourable for comammox and AOA over AOB due to the higher affinities most AOA and comammox have for ammonia [32]. Nitrite did not accumulate in any of the microcosms in this study, suggesting that nitrite oxidation was not a rate-limiting step under the tested conditions. There may be other edaphic factors that were not tested in this current study but could affect the growth and activity of these nitrifiers.

In summary, AOA and comammox were present in the alkaline forest soil, but there was no evidence of increase in the abundance of these microorganisms during the 30-day incubations nor of the effects the tested variables (temperature, moisture content, and ammonia concentration) had on them. This study also raises interesting questions about the role of turnover of microbial biomass and conditions where low or near-zero growth coincide with metabolic activity, which warrant further future investigation in nitrification research. In particular, RNA-based approaches and the use of isotopic tracers can facilitate the future research to explore the transcriptional activity and process rates of different nitrifiers in alkaline soils. In this study, we observed that low-abundance AOB respond rapidly to ammonia addition in a natural, previously unfertilized alkaline soil. This corroborates previous studies predominantly focused on agricultural, fertilized alkaline soil ecosystems, which indicated that AOB have an important role in alkaline soils.

## Supplementary Material

Supplementary_Material_240424_ycae093

## Data Availability

The datasets generated and analysed during the current study have been deposited in NCBI under Bioproject accession number PRJNA1063581 or are available from the corresponding author on reasonable request.
