# Supplementary material for: Investigating microbial and environmental drivers of nitrification in alkaline forest soil
Source: ISME Commun. 2024 Jul 11;4(1):ycae093. doi: 10.1093/ismeco/ycae093 (PMC11310595; doi:10.1093/ismeco/ycae093)
Supplement: Supplementary_Material_240424_ycae093 [file supplementary_material_240424_ycae093.docx]

**Supplementary Figure 1.** Ammonium consumption and nitrite + nitrate (NO_x_) production in 30 g ammonia amended microcosms to determine the optimal frequency of ammonium addition to the microcosms. A. Soil amended with 20 mg NH_4_^+^ -N kg^-1^ soil d.s.w. B. Soil amended with 200 mg NH_4_^+^ -N kg^-1^ soil d.s.w. Error bars represent the standard error of the mean (n=3).

**Supplementary Figure 2.** Phylogenetic tree of the 16S rRNA genes of the selected ammonia oxidising archaea (364 bp alignment) constructed using the Maximum Likelihood method with 100 bootstrap replicates. The tree was rooted using a sequence from *Saccharolobus solfataricus*, which does not belong to ammonia oxidising archaea. The accession numbers are indicated in the brackets.

**Supplementary Figure 3.** Phylogenetic tree of the 16S rRNA genes of the selected representatives of the genus *Nitrospira* (262 bp alignment) constructed using the Maximum Likelihood method with 100 bootstrap replicates. The tree was rooted using a sequence from *Nitrosomonas europaea*, which does not belong to genus *Nitrospira*. The accession numbers are indicated in the brackets.

**Supplementary Figure 4.** Phylogenetic tree of the 16S rRNA genes of the selected beta-proteobacteria (259 bp alignment) constructed using the Maximum Likelihood method with 100 bootstrap replicates. The tree was rooted using a sequence from *Nitrosococcus oceani*, which does not belong to beta-proteobacteria. The accession numbers are indicated in the brackets.

 **Supplementary Figure 5.** Phylogenetic tree of the 16S rRNA genes of the selected gamma-proteobacteria (261 bp alignment) constructed using the Maximum Likelihood method with 100 bootstrap replicates. The tree was rooted using a sequence from *Nitrosomonas europaea*, which does not belong to gamma-proteobacteria. The accession numbers are indicated in the brackets.

**Supplementary Table 1.** Primers used in this study.

| **Table S1. Primers used in this study** | | | | | |
| --- | --- | --- | --- | --- | --- |
| Primer name | Target microorganisms | Target | Sequence (5’- 3’) | Uses | Reference |
| 771F | Thaumarchaeota | 16S rRNA | ACGGTGAGGGATGAAAGCT | qPCR | 56 |
| 957R | Thaumarchaeota | 16S rRNA | CGGCGTTGACTCCAATTG | qPCR | 56 |
| A109F | Thaumarchaeota | 16S rRNA | ACKGCTCAGTAACACGT | PCR | 59 |
| 1492R | Universal | 16S rRNA | GYYACCTTGTTACGACTT | PCR | 60 |
| 1F | AOB | *amoA* | GGGGTTTCTACTGGTGGT | qPCR | 57 |
| 2R | AOB | *amoA* | CCCCTCKGSAAAGCCTTCTTC | qPCR | 57 |
| NEamoA1F | *Nitrosomonas europaea* | *amoA* | CCAGAGTGGGGTACCTTAGA | PCR | This study |
| NEamoA1R | *Nitrosomonas europaea* | *amoA* | TCCAATGTTACTGTCATGGGT | PCR | This study |
| comamoAF | Comammox | *amoA* | AGGNGAYTGGGAYTTCTGG | PCR, qPCR | 58 |
| comamoAR | Comammox | *amoA* | CGGACAWABRTGAABCCCAT | PCR, qPCR | 58 |
